# Clinical Outcomes of Small Incision Lenticule Extraction with Accelerated Cross-Linking (ReLEx SMILE Xtra) in Patients with Thin Corneas and Borderline Topography

**DOI:** 10.1155/2015/263412

**Published:** 2015-06-28

**Authors:** Sri Ganesh, Sheetal Brar

**Affiliations:** Nethradhama Superspeciality Eye Hospital, 256/14 Kanakapura Main Road, 7th Block, Jayanagar, Bangalore 560082, India

## Abstract

*Purpose*. To study the safety and clinical outcomes of ReLEx SMILE with accelerated cross-linking in individuals with thinner corneas, borderline topography, and higher refractive errors. *Methods*. Eligible patients first underwent SMILE procedure for correction of myopic refractive error. Following the removal of lenticule, 0.25% riboflavin in saline was injected into the interface and allowed to diffuse for 60 seconds. Finally, eye was exposed to UV-A radiation of 45 mW/cm^2^ for 75 seconds through the cap. Total energy delivered was 3.4 J/cm^2^. *Results*. 40 eyes of 20 patients with mean age of 26.75 ± 5.99 years were treated. Mean follow-up was 12 months ± 28.12 days. Mean spherical equivalent (SE) was −5.02 ± 2.06 D preoperatively and −0.24 ± 0.18 D postoperatively. The mean central corneal thickness (CCT) and keratometry changed from 501 ± 25.90 *µ*m to 415 ± 42.26 *µ*m and 45.40 ± 1.40 D to 41.2 ± 2.75 D, respectively. Mean uncorrected visual acuity (UCVA) was 20/25 or better in all eyes. No eyes lost lines of corrected distant visual acuity (CDVA). There were no complications like haze, keratitis, ectasia, or regression. *Conclusion*. Based on the initial clinical outcome it appears that SMILE Xtra may be a safe and feasible modality to prevent corneal ectasia in susceptible individuals.

## 1. Introduction

Refractive surgery in high myopia with thinner corneas and borderline topography is a challenging decision, with the most feared complication being regression and postoperative ectasia [1–4]. Collagen cross-linking (CXL) has been proven to be an effective modality to strengthen and stabilize the cornea in keratoconus and ectasia after corneal refractive surgery [5–11].

Another novel application of CXL is the combination of this technique with photo ablative procedures such as LASIK and PRK [12, 13], which has improved safety and better outcomes in subjects that were not suitable for corneal refractive correction due to their corneas being borderline. One such technique is LASIK Xtra which has been suggested as a safe and effective procedure for prophylaxis against postoperative ectasia in susceptible patients [13].

ReLEx SMILE is a flapless, all femtosecond laser technique which involves creation of a refractive lenticule with femtosecond laser and its removal from a small incision. This procedure has significant advantages over LASIK as there are no flap related complications, faster visual recovery, better long-term biomechanical stability, less postoperative dry eye, and fewer induction of aberrations [14]. ReLEx SMILE, when combined with collagen cross-linking intraoperatively, may further prevent the risk of future ectasia in susceptible individuals. Hence, this study was undertaken to observe the safety and visual outcomes following this procedure. The efficacy of the procedure in stabilizing the corneal biomechanics has not been studied. However, the one-year follow-up may establish safety.

## 2. Materials and Methods

The study was approved by Institutional Ethics Committee and adhered to the tenets of declaration of Helsinki. The nature of the procedure was explained and informed consent was obtained from all the patients participating in the study. Inclusion criteria were decided considering the assumed risk factors for ectasia based on published literature reports [15, 16].

According to the Randleman Scoring [16] for ectasia risk, eyes were classified into low (score < 2), moderate (score 3), and high risk (score ≥ 4). Eyes with moderate and high risk were selected for treatment; however, eyes with low risk, but associated with other risk factors as per published literature reports, were also included in the study [15].

Subjects with corneal thickness <450 um, established keratoconus, hyperopic refractive error, mixed astigmatism, concurrent eye infection, history of riboflavin allergy, past history of herpes infection or chemical injury, long-term topical or oral steroid use, and pregnant or nursing females were excluded from the study.

All patients underwent a thorough preoperative clinical examination including slit lamp biomicroscopy, dilated fundus examination, manifest and cycloplegic refraction, uncorrected (UDVA) and best corrected (CDVA) distant visual acuity using ETDRS charts at 4 m, applanation tonometry, topography (Orbscan IIz, Baush & Lomb), aberrometry (I Trace, Hoya), specular microscopy for endothelial cell count (ECD; Tomey EM-3000, Japan), contrast sensitivity (FACT), and dry eye assessment (Schirmer 1). Patients were examined on day 1, 1 month, 3 months, 6 months, and 12 months postoperatively. Recordings of UDVA and slit lamp examination for corneal state and any adverse effects were done on all visits. Postoperative anterior stromal haze was graded according to the scale described by Nakamura et al. [17]. On subsequent visits after day one, CDVA, topography, aberrometry, AS-OCT (Optovue), specular counts, contrast sensitivity, and dry eye assessment were also performed.

### 2.1. Surgical Technique

All patients underwent bilateral procedures by the same experienced surgeon (SG) on the same day. In the first stage, ReLEx SMILE was performed using standard surgical techniques. Visumax femtosecond laser (Carl Zeiss Meditec, Jena) was used to create a refractive lenticule with optical zone ranging from 6 to 6.5 mm, cap diameter 7–7.5 mm, and thickness of 100 *μ*m. The lenticule was then dissected and extracted through a 2 mm superior incision. Immediately after removal of the lenticule, 0.1 mL of Vibex Xtra, (Avedro, Waltham, MA) which is 0.25% riboflavin in saline was injected into the interface and allowed to diffuse for 60 seconds followed by washing of the interface with saline. Intraoperative slit lamp examination was done to ensure saturation of the cornea and the diffusion of dye into the anterior chamber. Using the Avedro KXL system (Waltham, MA), accelerated cross-linking with UV-A radiation was performed at 365 *μ*m wavelength, with energy of 45 mW/cm^2^ delivered in continuous mode to facilitate cross-linking through the cap for 75 seconds. Total energy delivered was 3.4 J/cm^2^. There were no intraoperative complications.

Postoperative medications included topical 0.3% Ofloxacin (Exocin, Allergan) 4 times for 3 days, 0.1% prednisolone acetate eye drops (Predforte, Allergen) in tapering dosages for 4 weeks, and lubricants 4–6 times for 4 weeks or more.

### 2.2. Statistical Analysis

Statistical analysis was performed using the SPSS software for Windows (IBM, Version 20.0.0). To analyze the data from preoperative to postoperative examinations and between consecutive postoperative visits, 1-way analysis of variance for repeated measures and paired *t*-tests were used. *P* value of <0.05 was considered significant. All values were expressed as mean ± SD.

## 3. Results

The study included 40 eyes of 20 patients of both genders. The baseline demographic and preoperative data is summarized in Table 1.

Of the 40 eyes, 17 (42.5%) eyes were classified as high risk (score of ≥4), 20 (50%) eyes as moderate risk (score of 3), and 3 (7.5%) eyes as low risk (score < 2) as per the Randleman ectasia risk score system. Eyes with low risk were treated as these patients had positive family history of keratoconus and a history of atopy.

At day 1 postoperatively, all eyes had clear corneas and the clarity was maintained till the last follow-up. Patients were comfortable and they did not report any significant symptoms. Mean follow-up was 12 months ± 28.12 days. There were no complications like postoperative keratitis, deep lamellar keratitis, epithelial ingrowth, ectasia, or regression in any of the eyes throughout the entire follow-up period. One month postoperatively, both eyes of one patient developed late mild haze (grade 2) and a drop in best-corrected visual acuity by one line which later recovered within three months after using topical steroids. This patient later elicited a history of autoimmune thyroiditis.

### 3.1. Visual Acuity, Safety, and Efficacy

Table 2 shows the visual acuity over time. There was a significant improvement in logMAR CDVA at 1 month and up to 3 months after surgery (*P* < 0.05), with no significant changes during the remainder follow-up (3 to 6 months and 6 to 12 months; *P* > 0.05).

There was significant improvement in logMAR BCVA at 1 month (*P* < 0.01), with no significant changes afterwards (*P* > 0.05).

**Figure 1:**
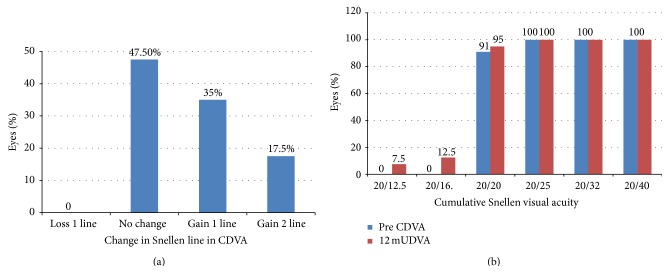
Change in Snellen lines of CDVA (CDVA: corrected distance visual acuity; SE: spherical equivalent) and cumulative Snellen visual acuity.

The CDVA was stable over time, with no eye losing visual acuity more than 1 line. At last visit, 19 eyes (47.5%) had no change in CDVA, 14 eyes (35%) gained 1 line, and 7 eyes (17.5%) gained 2 lines (Figure 1). Two eyes lost 1 line at one month follow-up, which improved to 20/20 visual acuity at 3 months. At 12 months, 38 eyes (95%) were 20/20 or better, while all (100%) eyes were 20/25 or better (Figure 1).

The overall mean efficacy index (postoperative UDVA/preoperative CDVA) and mean safety index (postoperative CDVA/preoperative CDVA) at the last follow-up visit were 1.03 and 1.29, respectively.

### 3.2. Stability and Predictability of Manifest Refraction

**Figure 2:**
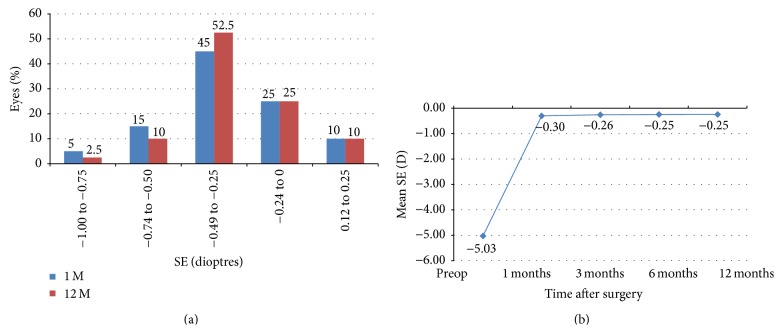
Change/stability of refraction in SE during the follow-up over time (*n* = 40 eyes) and distribution of postoperative SE (predictability). SE: spherical equivalent.

Figure 2 shows the improvement and stability of mean spherical equivalent (SE) over time. The mean SE decreased from −5.02 ± 2.06 D preoperative to −0.30 ± 0.22 D at 1 month, −0.24 ± 0.18 D at 6 months, and −0.24 ± 0.18 D at 1 year postoperatively.

**Figure 3:**
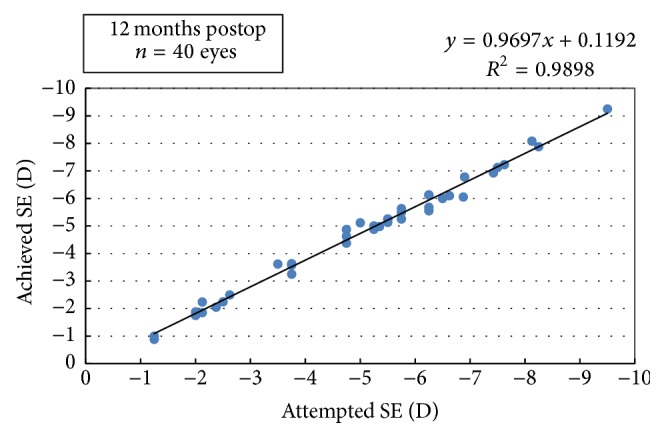
Intended versus achieved correction (manifest) at 12 months (SE: spherical equivalent).

High levels of predictability were achieved after surgery as 21 eyes (52.5%) were within ±0.25 D and 37 (92.5%) eyes were within ±0.5 D of the attempted correction at the 1-month visit. This improvement was maintained and remained stable over the subsequent postoperative follow-up. At 12 months, 26 eyes (65%) were within ±0.25 D and 39 eyes (97.5%) were within ±0.5 D of the target refraction. Undercorrection (SE −0.75 D or more) was detected in 2 eyes (5%) at 3 months, while all eyes were within ±0.75 D at 12 months (Figure 3).

### 3.3. Keratometry and Central Corneal Thickness

Table 3 depicts pre- and postoperative comparisons of keratometry and pachymetry over the complete follow-up period. The mean *K* readings from day one postoperative baseline to all postoperative time points were not statistically significant (*P* > 0.05).

**Figure 4:**
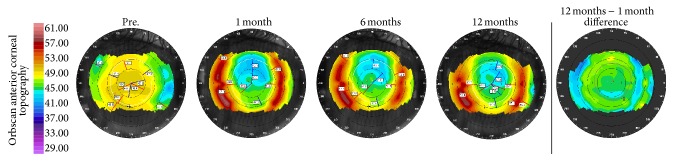
Orbscan anterior topography (Baush & Lomb) over 12-month postoperative course after SMILE Xtra for 26-year-old female with preoperative SE −7.62 D and CCT 478 *μ*m in right eye. (Randleman ectasia risk score: 4) (SE: spherical equivalent) (CCT: central corneal thickness).

Figure 4 shows serial anterior keratometry and difference maps of an eye treated with SMILE Xtra showing stability of keratometry over time.

**Figure 5:**
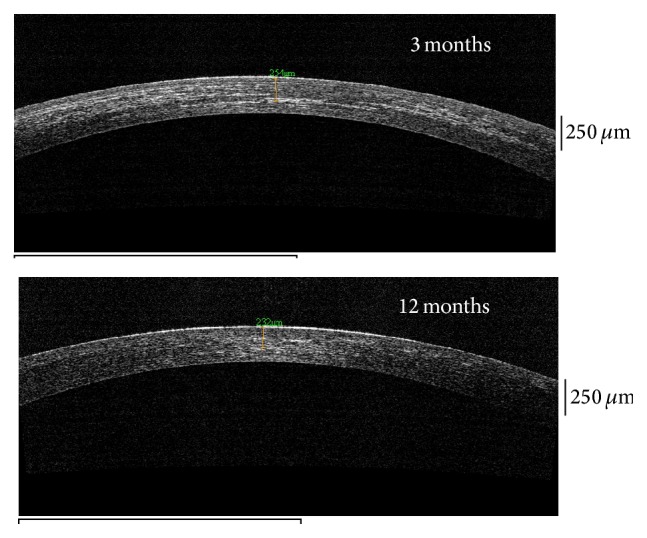
Anterior Segment Optical Coherence Tomography (AS-OCT) (Optovue, Fremont, CA) at 3 months and 12 months postoperative.

AS-OCT was done for all eyes postoperatively. The demarcation line was well defined at 1 month in 22 eyes (55%), 28 eyes (70%) at 3 months, 25 eyes (62.5%) at 6 months, and 12 eyes (30%) at 12 months (Figure 5).

In 4 eyes (10%) the demarcation line was absent. The average depth of stromal demarcation line was found to be 229.5 ± 19.1 *μ*m at 3 months and 225.7 ± 18.4 *μ*m at 6 months, after which the clarity decreased and was not appreciable in most of the eyes.

Table 4 shows ECD, HOA, and Schirmer 1 scores preoperatively and 12 months after SMILE Xtra. The mean endothelial cell density at last follow-up did not show a significant change from preoperative values (*P* = 0.22). HOA increased from 0.172 ± 0.08 *μ*m to 0.21 ± 0.08 *μ*m at 3 months and was 0.189 ± 0.03 *μ*m at the last follow-up, although the increase in HOA was statistically not significant at the last follow-up (*P* = 0.06). There was no significant difference in the Schirmer 1 scores pre- and postoperatively (*P* > 0.05).

### 3.4. Contrast Sensitivity

**Figure 6:**
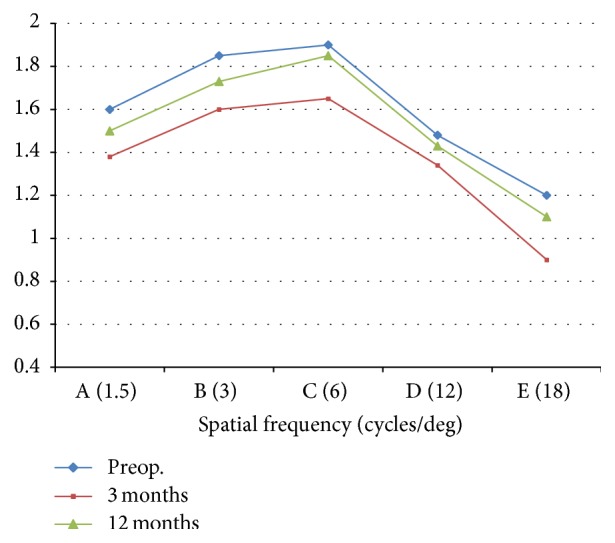
Contrast sensitivity (FACT) at 3 months and 12 months compared to preoperative.

Figure 6 shows the photopic contrast sensitivity over time. Although there was a drop in the contrast for all spatial frequencies, it remained in the normal acceptable range and showed a trend towards recovery in all subjects at the end of the mean follow-up. *P* was <0.01 for all spatial frequencies on all postoperative visits.

## 4. Discussion

Photorefractive keratectomy (PRK) and laser in situ keratomileusis (LASIK) have been recognized as weakening the corneal structural integrity by 14% to 33% and may increase the risk of ectasia [18–20]. To address this issue, adjuvant methods using collagen cross-linking at the time of laser refractive surgery have been suggested. For prophylactic purposes virtually any corneal excimer laser patient can be treated with cross-linking, although certain susceptible patients may benefit more [13]. The combination of both PRK and LASIK with accelerated cross-linking has come into practice indicating favourable outcomes [12, 13, 21], thus broadening the inclusion criteria for potential patients who were initially ineligible for excimer laser correction. Continuing with the series, we went one step ahead with a new procedure SMILE Xtra, which involves simultaneous high fluence cross-linking of cornea following ReLEx SMILE for myopia, in individuals who may be at risk of future corneal ectasia.

The concept is similar to the study by Kanellopoulos who did CXL in a femtosecond laser created corneal pocket in early keratoconus, suggesting a safe and effective alternative to conventional collagen cross-linking with advantages of no deepithelisation, faster healing, and reduced chances of infections and better patient comfort [22].

A recent study, combined accelerated cross linking with ReLEx SMILE in early keratoconus eyes, and one year follow-up suggested this a promising modality in arresting ectasia [23]. While previous studies highlighted cross-linking in corneal pocket to be a novel and epithelial sparing technique for achieving desired results on corneal stability in early keratoconus, our study focused on otherwise normal eyes, which could be at future risk of developing ectasia after ReLEx SMILE. Hence, our methodology differed from previous studies in several ways.

Although the efficacy of the classical Dresden protocol as well as accelerated cross-linking is established in long-term follow-up studies in keratoconus [7, 9, 10, 24], the prophylactic dose required in nonkeratoconus individuals who may be at risk of ectasia is not yet established. Review of the literature reveals that there is no standard protocol addressing such patients at risk, and different authors have suggested different regimens. When combined with LASIK, researchers have used high fluence of 30 mW/cm^2^ for variable durations, delivering energy ranging from 1.8 to 5.4 J/cm^2^, and found all regimens to be safe and effective [13, 21, 25]. Thus, there is wide variation and the minimum amount of energy to make the cornea sufficiently stiff to prevent ectasia in individuals at risk is still not known.

In our protocol, using the Avedro KXL device, we took advantage of the highest fluence at 45 mW/cm^2^ and accelerated cross-linking for 75 seconds, thus delivering a surface dose of 3.4 J/cm^2^ at the corneal plane. Since the cross-linking is facilitated through the cap, with the epithelium being intact, we proposed that the high fluence of 45 mW/cm^2^ would allow sufficient radiation to reach the stroma for effective cross-linking, since the epithelium itself may absorb substantial amount of UV-A radiation [26]. With a prophylactic procedure, we aimed at achieving an optimal energy, which is neither too high as is used for progressive keratoconus (>5.4 J/cm^2^) nor too low to be effective. We exploited the highest fluence of the system and found our regimen to be safe as it did not result in punctate keratitis, epithelial defects, deep lamellar keratitis, or endothelial toxicity.

We used VibeX Xtra (0.25% in saline) which is recommended for intrastromal application as it rapidly achieves high concentrations in the stroma. Since it is devoid of dextran, the diffusion into deeper layers is achieved as early as 60 seconds after application. This helps in selective placement of riboflavin in the stroma so that it absorbs and activates UVA light and achieves cross-linking without posing a threat to the underlying vital structures due to any stray radiation.

The stromal demarcation line was seen at a mean depth of 229.5 ± 19.1 *μ*m at 3 months which is significantly higher compared to the conventional cross-linkage in which it is typically seen at 300 *μ*m as early as two weeks [27]. Our results were consistent with the observations of Kymionis et al., who have recently shown that stromal demarcation line after accelerated cross-linkage was significantly higher than conventional cross-linking [28]. In our series, the mean depth of demarcation line was seen at even higher levels than that observed by Kymionis et al. The possible explanation could be due to differences in the total duration of exposure and amount of energy delivered which was lower (3.4 J/cm^2^) in our study as compared to theirs (5.4 J/cm^2^) because we did not intend to give the full therapeutic dose as is recommended for progressive keratoconus [5].

In SMILE Xtra, the dye gets rapidly diffused in both the overlying cap and the underlying stroma, theoretically resulting in effective cross-linking of both, whereas when combined with LASIK, the dye is administered only to the residual stromal bed and any contact with flap is avoided due to risk of postoperative microcicatricial changes leading to flap-interface disparity, flap wrinkling, irregular cross-linking, and difficulty in lifting the flap for retreatments. Due to this, cross-linking of the flap may not be very effective and may not offer security against flap dislocations in future.

SMILE is a flapless and a more tissue saving procedure as it takes similar or less tissue per diopter (~13 *μ* versus ~17 *μ* for LASIK) and the cap thickness contributes to the residual bed thickness thus allowing higher corrections to be safely achieved. Hence, it may not be unreasonable to say that even patients, ineligible for LASIK Xtra, may safely qualify to undergo corneal refractive correction with SMILE Xtra without increasing the risk of corneal destabilization. Due to this advantage, corrections using this technique may be safely attempted in early keratoconus with judicious patient selection.

We observed the refractive and topographic stability of the procedure to be excellent and well maintained at one year. However, corneal thickness in this series showed a statistically significant decrease from 1 month to 3 months postoperatively. The possible causes to this result could be a small sample size and compaction of stroma as a result of simultaneous accelerated cross linking, which appear to stabilize by 6 months after surgery.

None of the treated eyes had regression or evidence of postoperative ectasia clinically or topographically. No eye had loss of CDVA and did not require glasses or enhancement. Dry eye and the drop in contrast sensitivity was minimal, with near recovery of both transient problems by six months.

Initial results suggest that it is safe to combine ReLEX SMILE with accelerated cross-linking for future applications to prevent ectasia in susceptible individuals. It also establishes the safety of our regime of using 45 mW/cm^2^ UV irradiance and we believe there is no previous literature supporting this. It has been shown that the biomechanics with SMILE are better than LASIK [29] and the incidence of ectasia after SMILE may even be rare; however, we do not recommend this procedure for all patients undergoing SMILE routinely.

Eyes with moderate to high risk (Randleman score > 3) with or without additional risk factors may be offered this combined treatment.

Patients at risk should be identified and offered KXL as an adjunct to ensure long-term stability of results after SMILE. One limitation of our study was that due to the lack of equipment we could not study the changes in biomechanics and effect of the very high fluence cross-linking upon keratocyte activity with confocal microscopy. Also the superiority of this method compared to SMILE alone should be investigated through a comparative study.

Since the technique aims at future prophylaxis, longer follow-ups of these patients with studies on corneal biomechanics and the effects on corneal stabilization are required to establish the stability and efficacy of the procedure in preventing ectasia as compared to conventional LASIK Xtra.

## Figures and Tables

**Table 1:** Preoperative data (*n* = 40 eyes) who underwent SMILE Xtra.

Preop. parameters	Mean ± SD
Mean age (years)	26.75 ± 5.99 range (20–40)
Female : male	13 : 7
Mean CDVA (logMAR)	0.038 ± 0.06
SE (D)	−5.02 ± 2.06
*K* mean	45.4 ± 1.4 D
CCT (*μ*)	501.3 ± 25.9
HOA, RMS (*μ*)	0.172 ± 0.08
ECD (cells/mm^2^)	2872.0 ± 218.3
Schirmer I (mm)	34.2 ± 2.6

CDVA: Corrected Distant Visual Acuity, SE: spherical equivalent, *K*: keratometry, CCT: central corneal thickness, HOA: higher order aberrations, RMS: Root Mean Square, and ECD: endothelial cell density.

**Table 2:** Visual acuity and refraction during the complete postoperative follow-up.

Parameter Mean ± SD	1 month	3 months	6 months	12 months
UDVA (logMAR)	0.058 ± 0.07	0.023 ± 0.06	0.020 ± 0.06	0.020 ± 0.06

*P* value	Pre. versus 1 m *P* = 0.00	1 m versus 3 m *P* = 0.00	3 m versus 6 m *P* = 0.32	6 m versus 12 m *P* = 1.23

CDVA (logMAR)	−0.027 ± 0.09	−0.04 ± 0.07	−0.055 ± 0.06	−0.073 ± 0.081

*P* value	Pre. versus 1 m 0.00	1 m–3 m 0.40	3 m–6 m 0.24	6 m–12 m 0.07

Residual SE (D)	−0.30 ± 0.22	−0.26 ± 0.30	−0.24 ± 0.28	−0.24 ± 0.29

*P* value	Pre. versus 1 m 0.00	1 m–3 m 0.10	3 m–6 m 0.10	6 m–12 m 0.72

UDVA: uncorrected distant visual acuity, CDVA: corrected distant visual acuity, and SE: spherical equivalent.

**Table 3:** Changes in keratometry and central corneal thickness over time.

Mean + SD	Pre.	1 m	*P* value Pre. 1 m	3 m	*P* value 1 m–3 m	6 m	*P* value 3 m–6 m	12 m	*P* value 6 m–12 m
Keratometry (Orbscan II)	45.4 ± 1.4	41.2 ± 2.13	0.00	41.3 ± 2.52	0.16	41.2 ± 2.36	0.189	41.2 ± 2.75	0.183

CCT (Sirius)	501 ± 25.90	414.8 ± 41.33	0.00	416.2 ± 42.84	0.02	416.8 ± 42.66	0.256	415 ± 42.26	0.07

CCT: central corneal thickness; Keratometry done using Orbscan II; CCT measured using Sirius topography system; *P* value calculated with repeated measures of ANOVA.

**Table 4:** ECD, higher order aberrations (HOA) at 6 mm pupil size and Schirmer 1 scores after SMILE Xtra (*n* = 40 eyes).

(Mean ± SD)	Preop.	12 months	*P* value^*∗*^
ECD (cells/mm^2^)	2695.13 ± 222.8	2682.5 ± 231.8	0.22

HOA (RMS, *μ*)	0.172 ± 0.08	0.189 ± 0.09	0.06

Schirmer I (mm)	28.2 ± 2.1	28.5 ± 1.3	0.08

ECD: endothelial cell density, HOA: higher order aberrations, RMS: Root Mean Square, and ^*∗*^12 months versus preoperative (paired *t*-test).
